# Comparison of Emergency Department Use Between Pregnant People With and Without Disabilities in Ontario, Canada

**DOI:** 10.1001/jamanetworkopen.2023.27185

**Published:** 2023-08-03

**Authors:** Hilary K. Brown, Catherine Varner, Joel G. Ray, Natalie V. Scime, Kinwah Fung, Astrid Guttmann, Susan M. Havercamp, Simone N. Vigod, Yona Lunsky

**Affiliations:** 1 Department of Health and Society, University of Toronto Scarborough, Toronto, Ontario, Canada; 2 Dalla Lana School of Public Health, University of Toronto, Toronto, Ontario, Canada; 3 Women’s College Research Institute, Women’s College Hospital, Toronto, Ontario, Canada; 4 ICES, Toronto, Ontario, Canada; 5 Department of Family and Community Medicine, University of Toronto, Toronto, Ontario, Canada; 6 Mount Sinai Hospital, Toronto, Ontario, Canada; 7 Li Ka Shing Knowledge Institute, St Michael’s Hospital, Toronto, Ontario, Canada; 8 Hospital for Sick Children, Toronto, Ontario, Canada; 9 Edwin SH Leong Centre for Healthy Children, University of Toronto, Toronto, Ontario, Canada; 10 Department of Pediatrics, University of Toronto, Toronto, Ontario, Canada; 11 Nisonger Center, Wexner Medical Center, Ohio State University, Columbus; 12 Department of Psychiatry, University of Toronto, Toronto, Ontario, Canada; 13 Azrieli Adult Neurodevelopmental Centre, Centre for Addiction and Mental Health, Toronto, Ontario, Canada

## Abstract

**Importance:**

Emergency department (ED) use in pregnancy is common and occurs for a variety of reasons, including obstetrical complications, exacerbated underlying conditions, and inadequate outpatient health care access. People with disabilities have elevated rates of certain medical, psychiatric, and obstetrical conditions as well as inadequate access to prenatal care; their risk of ED use in pregnancy is not known, however.

**Objective:**

To compare the risk of ED use in pregnancy among people with physical, sensory, and intellectual or developmental disabilities with those without disabilities.

**Design, Setting, and Participants:**

Population-based cohort study leveraging linked administrative health data sets in Ontario, Canada, April 2003 to March 2019. Analysis included all recognized pregnancies to people with a preexisting physical, sensory, intellectual or developmental, or 2 or more (multiple) disabilities, and those without a disability. Data were analyzed from May 2022 to January 2023.

**Exposure:**

Disability was ascertained using algorithms applied to 2 or more outpatient physician visits or 1 or more ED visits or hospitalizations before conception.

**Main outcomes and measures:**

Modified Poisson regression–generated adjusted relative risks (aRR) and 95% CIs for any ED visit in pregnancy, from the estimated conception date up to the end of the pregnancy, adjusted for age, parity, income quintile, rurality, immigrant status, and preexisting chronic conditions, mental illness, and substance use disorders.

**Results:**

The cohort included 2 659 895 pregnant people with physical (221 739 participants; mean [SD] age, 29.8 [6.1] years), sensory (71 891 participants; mean [SD] age, 29.1 [6.4] years), intellectual or developmental (3877 participants; mean [SD] age, 26.1 [6.7] years), and multiple disabilities (14 359 participants; mean [SD] age, 29.5 [6.5] years), and pregnant people without a disability (2 348 023 participants; mean [SD] age, 29.4 [5.9] years). The rate of ED visits in pregnancy was 25.4% in people without a disability (596 771 visits). Relative to these individuals, the aRR for ED use was elevated in people with physical (aRR, 1.26; 95% CI, 1.25-1.27), sensory (aRR, 1.15; 95% CI, 1.14-1.17), intellectual or developmental (aRR, 1.33; 95% CI, 1.28-1.38), and multiple disabilities (aRR, 1.43; 95% CI, 1.40-1.46).

**Conclusions and Relevance:**

In this population-based study, people with disabilities were at elevated risk of ED use in pregnancy. This finding underscores the need for research on the benefits of proactive strategies to manage preexisting conditions in these individuals, improve their access to outpatient obstetrical and medical care, and prepare them for when ED visits occur.

## Key Points

**Question:**

Are individuals with disabilities at greater risk of emergency department use in pregnancy compared with those without disabilities?

**Findings:**

In a cohort study of more than 2 million pregnant people in Ontario, Canada, the adjusted relative risk of emergency department use was 26% higher in those with physical, 15% higher in those with sensory, 33% higher in those with intellectual or developmental, and 43% higher in those with multiple disabilities, compared with people without disabilities.

**Meaning:**

These findings suggest the need for research on the benefits of strategies to manage preexisting conditions and improve access to outpatient care for disabled individuals in pregnancy.

## Teaser

This cohort study compares emergency department use between pregnant people with and without disabilities in Ontario, Canada.

## Introduction

Pregnancy-related problems are the fourth leading indication for emergency department (ED) use in reproductive-aged women.^1^ ED use in pregnancy reflects a wide range of medical concerns, varying in their degree of urgency and including obstetric complications and exacerbated underlying conditions.^2,3^ There is evidence that one-third of ED visits in pregnancy (eg, for nausea and vomiting in early pregnancy) could be managed in nonemergent settings or prevented with outpatient health care and education, suggesting inadequate access to effective outpatient care plays a role in the use of the ED during this time.^2^ ED visits in pregnancy are stressful for families and inefficient for the health care system,^4^ so identifying at-risk groups and providing appropriate outpatient health care and other resources are of critical importance.

A number of populations are at high risk for ED use in pregnancy, including young parents, and those who experience poverty, mental illness, addiction, and chronic physical conditions.^3,5,6,7,8^ One group whose ED use in pregnancy has received less attention is people with disabilities. This is an important issue, since 13% of pregnancies are to people with a disability,^9^ and people with disabilities have elevated rates of known predictors of ED use in pregnancy, including poverty and chronic physical and mental health conditions.^10,11^ They also experience barriers accessing high-quality outpatient care, including insufficient clinician training, negative clinician attitudes, and inadequate accommodations related to mobility and communication needs.^12,13,14^ Yet, only 3 studies—all from the US, which has a multipayer health system—have examined ED use in pregnant people with disabilities.^15,16,17^ These studies showed elevated rates of ED visits in women with disabilities in pregnancy.^15,16,17^ There is a need for population-based studies from other health care systems, and with further details, such as the acuity of the reason for the ED visit and the outcome of the visit (eg, admission or follow-up care in the community). Such data are needed to inform strategies to prevent ED visits in pregnant people with disabilities when possible, and create supports for them in the ED and postdischarge.

In a population-based cohort in Ontario, Canada, we compared the risk of ED use in pregnancy in people with physical, sensory, and intellectual or developmental disabilities with those without disabilities. Among those with an ED visit, we also compared rates of hospital admission from the ED and postdischarge outpatient care in people with physical, sensory, and intellectual or developmental disabilities with those without disabilities.

## Methods

### Study Design and Data Sources

We undertook a population-based cohort study in Ontario, Canada, following the Strengthening the Reporting of Observational Studies in Epidemiology (STROBE) reporting guideline.^18^ Under Ontario’s universal health care plan, all essential health care services, including outpatient and hospital care, are provided at no direct cost to residents of the province. Administrative health data resulting from the use of these health care services were accessed and analyzed at ICES (formerly, the Institute for Clinical Evaluative Sciences) in Toronto. Databases with information on outpatient physician visits, ED visits, hospitalizations, and sociodemographic characteristics (eTable 1 in Supplement 1) were linked using a unique encoded identifier. ICES administrative health data have been shown to be accurate and complete.^19^

ICES is a prescribed entity under Ontario’s Personal Health Information Protection Act (PHIPA). Section 45 of PHIPA authorizes ICES to collect personal health information, without consent, for the purpose of analysis or compiling statistical information with respect to the management, evaluation, or monitoring of the allocation of resources to or planning for all or part of the health system. Projects that use data collected by ICES under section 45 of PHIPA, and use no other data, are exempt from research ethics board review. The use of the data in this project was authorized under section 45 and approved by ICES’ Privacy and Legal Office.

### Study Population

The study population included recognized pregnancies conceived between April 1, 2003, and March 31, 2019, to individuals aged 49 years who were eligible for Ontario’s health insurance plan in the 2 years before conception. A recognized pregnancy included a livebirth at 20 or more weeks, stillbirth at 20 or more weeks, miscarriage at less than 20 weeks, induced abortion at any gestational age, or threatened abortion (vaginal bleeding at <20 weeks, or unspecified hemorrhage without a later recognized pregnancy outcome) (eTable 2 in Supplement 1).^6^ Inclusion of threatened abortion allowed us to capture pregnancies that might have ended in miscarriage without a health care encounter, and would have otherwise been missed.^6^ We estimated conception dates as follows: for livebirths and stillbirths, gestational age in the birth record^20^ was subtracted from the delivery date. For miscarriages, induced abortions, and threatened abortions where gestational age was missing, we estimated gestational ages as 8, 9, and 10 weeks, respectively, according to median values,^6^ and subtracted these from the pregnancy’s end date. The cohort was then divided into pregnancies to people with and without a disability. Disabilities were identified using algorithms developed for health administrative data.^21,22,23^ Consistent with prior research,^22,23,24^ we categorized a pregnant individual as having a disability if 2 or more physician visits or 1 or more ED visits or hospitalizations between database inception and conception contained a diagnostic code for a physical (congenital anomaly, musculoskeletal disorder, neurological disorder, or permanent injury), sensory (hearing loss or vision loss), intellectual or developmental (autism spectrum disorder, chromosomal anomalies resulting in intellectual disability, or fetal alcohol spectrum disorder), or 2 or more disabilities (multiple disabilities) (eTable 3 in Supplement 1). Pregnant individuals without a disability were the referent group.

### Outcomes

Our primary outcome was any ED visit from conception to the end of the pregnancy. ED visits were identified in the National Ambulatory Care Reporting System data set, which captures data on unscheduled visits by patients who may need immediate care to facilities staffed by physicians 24 hours per day, 7 days per week.^6^ As secondary outcomes, we examined these visits by: (1) number (0, 1, 2, or ≥3); (2) primary discharge diagnosis type (obstetric, other medical, or psychiatric); and (3) Canadian Triage Association Score (CTAS), used to indicate how urgently a patient needs to be seen and the most appropriate treatment area or monitoring level (high acuity, CTAS 1-2; moderate acuity, CTAS 3; low acuity, CTAS 4-5).^25^ We recorded the proportion of first ED visits occurring at each week of gestation (livebirths only). Finally, we examined the proportion of people with an ED visit (1) who were admitted to hospital from the ED and (2) of those not admitted, who had care with their obstetrician or primary care physician within 7 and 14 days.^26^

### Covariates

We measured several confounders: age, parity, neighborhood income quintile, rurality, immigrant or refugee status, and comorbidities. Neighborhood income quintile was measured by linking postal codes to Census income data. Rurality was measured using the Rurality Index of Ontario.^27^ Immigrants, refugees, and long-term residents (ie, who were born in or migrated to Ontario before 1985) were identified using the Immigrants, Refugees, and Citizenship Canada Permanent Residents Database. Stable and unstable chronic conditions in the 2 years before conception were measured using the Johns Hopkins Adjusted Clinical Groups System version 10 collapsed ambulatory diagnostic groups (wherein disability diagnoses were removed to avoid overlap).^28^ Mental illness (ie, mood or anxiety, psychotic, or other mental disorders) and substance use disorders (ie, drug or alcohol use disorders) were identified using diagnoses in 2 or more physician visits or 1 or more ED visits or hospitalizations in the 2 years before conception.

We also measured several indicators of outpatient health care use. Continuity of primary care was defined as the proportion of primary care visits made to the regular primary care physician in the 2 years before conception among individuals with 3 or more visits during this period, classified as low (0.0-0.50), moderate (0.51-0.80), or high (>0.80),^29^ with infrequent users (<3 visits) as a separate group. Prenatal care adequacy was measured using the Revised Graduated Prenatal Care Utilization Index,^30^ which uses timing of initiation of prenatal care, gestational age, and total number of prenatal care visits to identify intensive, adequate, inadequate or intermediate, and no or unknown prenatal care. The type of physician providing the majority of prenatal care was classified as obstetrician, family physician, shared care, and other/none.

### Statistical Analysis

We used frequencies and percentages to describe the cohort’s characteristics. Due to our large cohort, we used standardized differences to compare each disability group to those without a disability, since, unlike *P* values, standardized differences are not associated with sample size.^31^

We then used modified Poisson regression,^32^ with generalized estimating equations to account for the presence of multiple pregnancies per individual in the study period,^33^ to examine the relative risk (RR) of any ED visit in pregnancy comparing people with physical, sensory, intellectual or developmental, and multiple disabilities with those without a disability. The adjusted models included age, parity, income quintile, rurality, immigrant status, stable and unstable chronic conditions, mental illness, and substance use disorder. We considered health care use variables to be pathway variables and did not include them in the main multivariable models.

In additional analyses, we repeated the main model adding the health care use variables. We used multinomial logistic regression to examine odds ratios of 1, 2, or 3 or more ED visits in pregnancy vs 0. We used modified Poisson regression to examine any ED visit in pregnancy by discharge diagnosis and triage acuity. For live births, we reported the proportion of first ED visits occurring at each gestational week. To determine if ED use patterns differed with medical and psychiatric history or pregnancy outcome, we repeated the main analyses, stratifying the models by comorbidity status (0 or ≥1 preexisting chronic condition, mental illness, or substance use disorder) and pregnancy type (livebirth, stillbirth, induced abortion, miscarriage, or threatened abortion). Finally, we examined the proportion of people with an ED visit in pregnancy who were then admitted to the hospital and, among those not admitted, the proportion who received care with their obstetrician or primary care physician within 7 and 14 days of the ED visit. Analyses used SAS version 9.4 (SAS Institute) and were conducted from May 2022 to January 2023.

## Results

The cohort included pregnant people with physical (221 739 participants; mean [SD] age, 29.8 [6.1] years), sensory (71 891 participants; mean [SD] age, 29.1 [6.4] years), intellectual or developmental (3877 participants; mean [SD] age, 26.1 [6.7] years), and multiple (14 359 participants; mean [SD] age, 29.5 [6.5] years) disabilities, and pregnant people without a disability (2 348 023 participants; mean [SD] age, 29.4 [5.9] years) (Table 1). Compared with people without a disability, those with intellectual or developmental disabilities were younger and more likely to live in low-income neighborhoods. People in all disability groups were less likely to be immigrants or refugees. Those with physical and multiple disabilities were more likely to have stable chronic conditions, and all disability groups were more likely to have unstable chronic conditions. All disability groups were more likely to have a mental illness predating the pregnancy, while those with physical, intellectual or developmental, and multiple disabilities were more likely to have a substance use disorder. Individuals with physical and multiple disabilities were less likely to have infrequent primary care use, and those with intellectual or developmental disabilities were more likely to have inadequate prenatal care.

**Table 1.** Baseline Characteristics of Individuals Aged 15 to 49 years With a Physical, Sensory, Intellectual or Developmental, or Multiple Disabilities, and Those Without a Disability, Who Had a Recognized Pregnancy in Ontario, Canada, 2003 to 2019

Variable^a^	Pregnant people, No. (%)				
	Physical disability only (n = 221 739)	Sensory disability only (n = 71 891)	Intellectual or developmental disability only (n = 3877)	Multiple disabilities (n = 14 359)	No disability (n = 2 348 023)
Age, y					
Overall, mean (SD)	29.8 (6.1)	29.1 (6.4)	26.1 (6.7)^a^	29.5 (6.5)	29.4 (5.9)
15-24	44 690 (20.2)	18 277 (25.4)	1815 (46.8)^a^	3496 (24.3)	502 833 (21.4)
25-34	126 634 (57.1)	38 858 (54.1)^a^	1574 (40.6)^a^	7492 (52.2)^a^	1 382 474 (58.9)
35-49	50 415 (22.7)	14 756 (20.5)	488 (12.6)^a^	3371 (23.5)	462 716 (19.7)
Multiparous	95 717 (43.2)	29 071 (40.4)	1609 (41.5)	6134 (42.7)	986 359 (42.0)
Neighborhood income					
Quintile 1 (lowest)	48 310 (21.8)	16 007 (22.3)	1376 (35.5)^a^	3686 (25.7)	527 798 (22.5)
Quintile 2	44 920 (20.3)	14 719 (20.5)	872 (22.5)	2929 (20.4)	479 777 (20.4)
Quintile 3	45 467 (20.5)	14 639 (20.4)	679 (17.5)	2843 (19.8)	483 591 (20.6)
Quintile 4	45 615 (20.6)	14 733 (20.5)	482 (12.4)^a^	2684 (18.7)	471 664 (20.1)
Quintile 5 (highest)	36 522 (16.5)	11 547 (16.1)	433 (11.2)^a^	2159 (15.0)	376 538 (16.0)
Missing	905 (0.4)	246 (0.3)	35 (0.9)	58 (0.4)	8655 (0.4)
Rural region of residence					
Rural	26 748 (12.1)	7321 (10.2)	413 (10.7)	1675 (11.7)	217 128 (9.2)
Urban	194 820 (87.9)	64 515 (89.7)	3451 (89.0)	12 665 (88.2)	2 129 343 (90.7)
Missing	171 (0.1)	55 (0.1)	13 (0.3)	19 (0.1)	1552 (0.1)
Immigrant or refugee	29 277 (13.2)^a^	11 544 (16.1)^a^	271 (7.0)^a^	1431 (10.0)^a^	616 300 (26.2)
Stable chronic conditions	55 868 (25.2)^a^	16 806 (23.4)	847 (21.8)	4387 (30.6)^a^	478 693 (20.4)
Unstable chronic conditions	34 585 (15.6)^a^	9985 (13.9)^a^	551 (14.2)^a^	3101 (21.6)^a^	252 280 (10.7)
Mental illness	54 250 (24.5)^a^	15 535 (21.6)^a^	1789 (46.1)^a^	4589 (32.0)^a^	381 912 (16.3)
Substance use disorder	6074 (2.7)^a^	1304 (1.8)	405 (10.4)^a^	664 (4.6)^a^	31 296 (1.3)
Continuity of primary care					
Low (0.0-0.50)	72 517 (32.7)	23 654 (32.9)	1287 (33.2)	4797 (33.4)	751 041 (32.0)
Moderate (0.51-0.80)	57 290 (25.8)	18 008 (25.0)	915 (23.6)	3741 (26.1)	561 307 (23.9)
High (>0.80)	46 084 (20.8)	13 844 (19.3)	650 (16.8)	3056 (21.3)	439 735 (18.7)
Infrequent users (<3 visits)	45 848 (20.7)^a^	16 385 (22.8)	1025 (26.4)	2765 (19.3)^a^	595 940 (25.4)
Prenatal care adequacy					
Intensive	60 023 (27.1)	18 601 (25.9)	1008 (26.0)	4475 (31.2)^a^	556 658 (23.7)
Adequate	65 499 (29.5)	21 503 (29.9)	985 (25.4)^a^	3892 (27.1)	731 376 (31.1)
Inadequate/intermediate	40 280 (18.2)	13 324 (18.5)	907 (23.4)^a^	2591 (18.0)	436 988 (18.6)
None/unknown	55 937 (25.2)	18 463 (25.7)	977 (25.2)	3401 (23.7)	623 001 (26.5)
Type of prenatal care clinician					
Obstetrician only	58 467 (26.4)	19 509 (27.1)	1055 (27.2)	3954 (27.5)	622 676 (26.5)
Family physician only	39 242 (17.7)	12 305 (17.1)	697 (18.0)	2406 (16.8)	401 375 (17.1)
Shared care	75 411 (34.0)	23 742 (33.0)	1278 (33.0)	5199 (36.2)	763 781 (32.5)
None/unknown	48 619 (21.9)	16 335 (22.7)	847 (21.8)	2800 (19.5)^a^	560 191 (23.9)

^a^ Standardized difference greater than 0.10 comparing people with a given disability vs people without any disability.

ED visits in pregnancy were common in people without a disability (596 771 participants [25.4%]), but they were more likely to occur in those with physical (76 594 participants [34.5%]; RR, 1.34; 95% CI, 1.33-1.35), sensory (22 421 participants [31.2%]; RR, 1.22; 95% CI, 1.20-1.23), intellectual or developmental (1777 participants [45.8%]; RR, 1.75; 95% CI, 1.68-1.83), and multiple (6041 participants [42.1%]; RR, 1.62; 95% CI, 1.58-1.65) disabilities. These associations remained statistically significant even after covariate adjustment (physical aRR, 1.26; 95% CI, 1.25-1.27; sensory aRR, 1.15; 95% CI, 1.14-1.17; intellectual or developmental aRR, 1.33; 95% CI, 1.28-1.38; multiple aRR, 1.43; 95% CI, 1.40-1.46) (Table 2). Results were unchanged after adding health care use variables to the model (eTable 4 in Supplement 1).

**Table 2.** Risk of Any Emergency Department Visit During Pregnancy, Comparing Individuals With Various Types of Disabilities With Those Without a Disability

Disability type	Individuals with outcome, No. (%)	Unadjusted RR (95% CI)	Adjusted RR (95% CI)^a^
No disability	596 771 (25.4)	1 (Reference)	1 (Reference)
Physical only	76 594 (34.5)	1.34 (1.33-1.35)	1.26 (1.25-1.27)
Sensory only	22 421 (31.2)	1.22 (1.20-1.23)	1.15 (1.14-1.17)
Intellectual or developmental only	1777 (45.8)	1.75 (1.68-1.83)	1.33 (1.28-1.38)
Multiple	6041 (42.1)	1.62 (1.58-1.65)	1.43 (1.40-1.46)

Abbreviation: RR, relative risk.

^a^ Adjusted for age, parity, neighborhood income quintile, region of residence, immigrant status, stable and unstable chronic conditions, mental illness, and substance use disorders.

**Figure 1.**
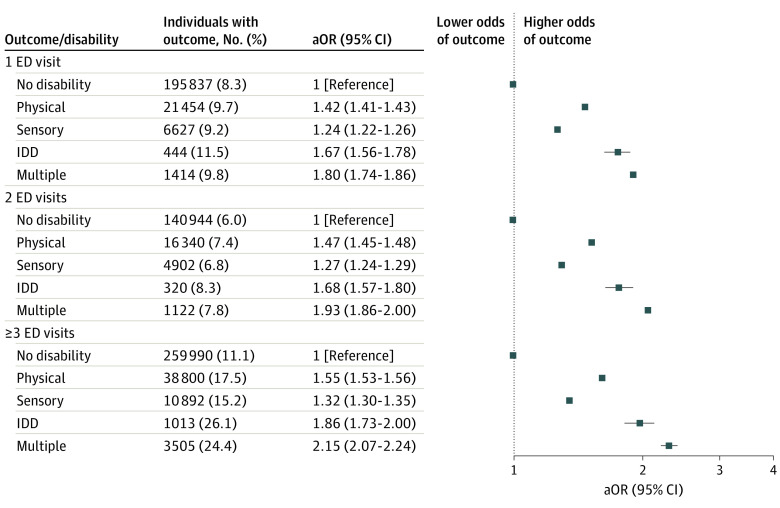
Odds of Having 1, 2, or 3 or More Emergency Department (ED) Visits During Pregnancy, Comparing Individuals With Various Types of Disabilities With Those Without a Disability Adjusted model controls for age, parity, neighborhood income quintile, region of residence, immigrant status, stable and unstable chronic conditions, mental illness, and substance use disorders. aOR indicates adjusted odds ratio; IDD, intellectual or developmental disability.

**Figure 2.**
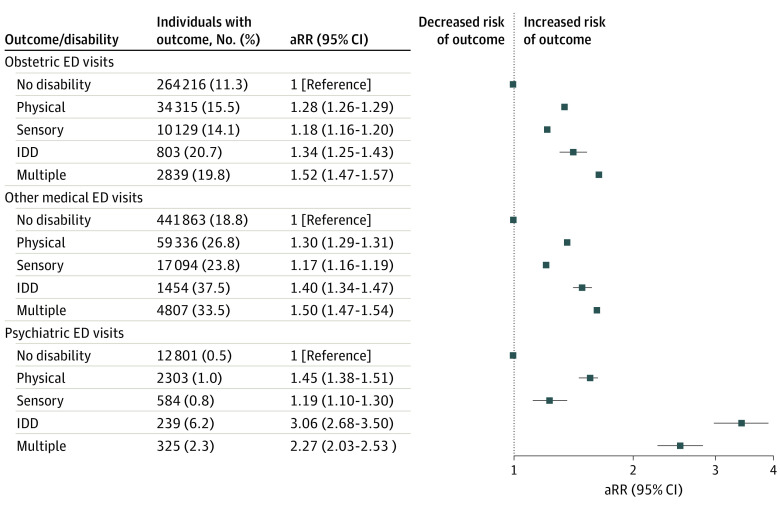
Risk of Any Emergency Department (ED) Visit During Pregnancy, Comparing Individuals With Various Types of Disability With Those Without a Disability, by Main Diagnosis Type at Discharge From the ED Adjusted model controls for age, parity, neighborhood income quintile, region of residence, immigrant status, stable and unstable chronic conditions, mental illness, and substance use disorders. aRR indicates adjusted relative risk; IDD, intellectual or developmental disability.

**Figure 3.**
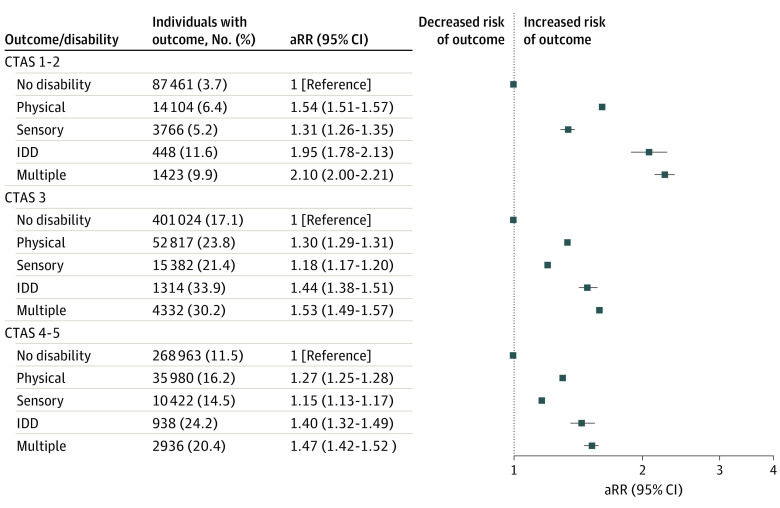
Risk of Any Emergency Department Visit During Pregnancy, Comparing People With Various Types of Disabilities With Those Without a Disability, by Canadian Triage Association Score (CTAS) Score High acuity is reflected by a CTAS score of 1 to 2, moderate acuity by a score of 3, and low acuity by a score of 4 to 5. Adjusted model controls for age, parity, neighborhood income quintile, region of residence, immigrant status, stable and unstable chronic conditions, mental illness, and substance use disorders. aRR indicates adjusted relative risk; IDD, intellectual or developmental disability.

Individuals in all disability groups were more likely to have 1 only, 2 only, and 3 or more ED visits in pregnancy (Figure 1). The association between disability and ED visits in pregnancy persisted irrespective of discharge diagnosis type (Figure 2) and triage acuity (Figure 3). Across all groups with and without disabilities with a livebirth, the temporal distribution of first ED visits showed the highest proportions of ED visits occurred in early pregnancy (eFigure in Supplement 1). The risks of ED visits in pregnancy were elevated in people with disabilities with and without preexisting comorbidities (eTable 5 in Supplement 1) and with a pregnancy ending in a livebirth, stillbirth, miscarriage, induced abortion, and threatened abortion (eTable 6 in Supplement 1).

Finally, among individuals with an ED visit in pregnancy, those with physical, intellectual or developmental, and multiple disabilities were more likely than individuals without a disability to be admitted to a hospital from the ED (eTable 7 in Supplement 1). In individuals with an ED visit that did not result in admission, those with disabilities had only slightly higher rates of visits with their obstetrician or primary care physician within 7 and 14 days of their ED visit (eTable 8 in Supplement 1).

## Discussion

In this population-based cohort study, rates of ED visits in pregnancy were high overall, and especially in people with disabilities. People with disabilities were at elevated risk of multiple ED visits in pregnancy, ED visits for not only obstetrical but also other medical and psychiatric conditions, and ED visits for high-acuity reasons resulting in hospital admission and low-acuity reasons. The highest rates of ED visits were in people with intellectual or developmental and multiple disabilities, with double to triple the risk, even after adjustment for multiple ED visits, ED visits with psychiatric diagnoses, and ED visits with high-acuity reasons. Together, these data signal a need for research on the value of proactive strategies to prevent ED visits, when possible, in pregnant people with disabilities, and to prepare them for when ED visits occur.

The only studies other than the current study to examine ED use in pregnant people with disabilities were based in the US multipayer health system. A study^17^ of Medicaid recipients in Florida, Georgia, New Jersey, and Texas found people with disabilities had 1.22 to 1.63 times the odds of ED visits in pregnancy compared with those without disabilities. One study^15^ from the Massachusetts Pregnancy to Early Life Longitudinal data system found higher rates of ED visits in pregnancy in people with disabilities compared with those without, with those with mental or comorbid mental and multiple physical diagnoses having the highest ED use. Another^16^ found people with intellectual or developmental disabilities had twice the odds of ED visits in pregnancy compared with people without these disabilities. Our data, from a universal health care system, are consistent with these studies. Our study also adds to the literature by showing higher rates of ED use in pregnancy in people with disabilities in a cohort of recognized pregnancies, and shows the consistency of this trend across important clinical factors, including diagnosis and acuity.

ED use in pregnancy reflects a range of concerns, including obstetric and medical complications and inadequate outpatient care access.^2,3^ Disabled people have elevated rates of pregnancy complications^23,24,34,35^ and comorbidities that may be exacerbated in pregnancy.^10,11^ In our cohort, rates of ED visits for obstetrical, other medical, and psychiatric reasons were higher in people with disabilities than those without. However, ED visits in pregnancy in people with disabilities may also reflect shortcomings in outpatient care. Although we could only measure the timing and number of prenatal visits,^36,37^ qualitative research shows people with disabilities are less likely to receive high-quality prenatal care, with such settings being physically inaccessible, lacking resources to address communication needs, and offering insufficient appointment times to address disability and pregnancy-related concerns.^12,13,14^ Clinicians may also have negative attitudes toward pregnant disabled people and insufficient knowledge of their health and social needs.^14^ Indeed, in addition to high-acuity visits, people with disabilities in our cohort had higher rates of low-acuity ED visits, suggesting some of their reasons for ED use could have been managed in outpatient settings. It is thus likely a combination of unavoidable and avoidable factors that cause pregnant people with disabilities to seek care in an ED setting at elevated rates.

### Strengths and Limitations

Strengths of our study include the population-based data source, long lookback period for measuring disability, and inclusive cohort of all recognized pregnancies. However, individuals with a pregnancy ending in miscarriage or induced abortion outside Ontario’s health system were not included. Gestational age for miscarriages, induced abortions, and threatened abortions had to be estimated according to median values,^6^ but findings were similar in pregnancies ending in a livebirth or stillbirth, for which gestational age data are complete.^20^ Use of diagnoses to ascertain disability reflects a medical model, without capturing the association of the environment with participation.^38^ Also, disability may have been misclassified if the clinician did not record diagnoses or if individuals did not access care for their disability. Such misclassification would likely bias risk estimates toward the null. Other than a broad measure of acuity, we were not able to classify ED visits according to whether they were preventable in an outpatient setting. We had no data on individual-level income, education level, experiences of racism, social support, or ED use in past pregnancies. We also had no data on the quality of outpatient prenatal care, which might be a more important contributor to observed disparities than the number and timing of visits.

ED use is a barometer of not only acute illness but also chronic illness and access to outpatient care. Our data suggest the need for research assessing the effectiveness of multifaceted strategies to prevent ED visits in pregnant people with disabilities and prepare them for when ED visits occur.^36^ For example, proactive preconception care and interdisciplinary prenatal care approaches could be beneficial to manage disability-related concerns and comorbidities in disabled people and reduce their risks of urgent ED visits due to exacerbated underlying conditions and obstetrical complications.^10,11^ There may also be a need for efforts to improve the capacity of outpatient services to provide accessible prenatal care, thus reducing risks of nonurgent ED visits. This will require better clinician training and collaboration with community services to address barriers associated with clinician knowledge and unmet access needs.^12,13,14^ High-quality prenatal education in community settings may also be critical for improving health literacy associated with urgent vs nonurgent concerns.^39^ However, given one-third to one-half of people with disabilities had an ED visit in pregnancy, there is a need for assessment of strategies to ensure that they and ED staff are prepared for such care, including patient care plans to facilitate communication with ED staff and outpatient clinicians.^40^ Collectively, these efforts are needed to address the high rate of ED use by pregnant people with disabilities.

## Conclusions

In a large, population-based study in Ontario, Canada, we found that people with disabilities were at elevated risk of ED use in pregnancy. Our data demonstrate the need for research on the benefits of preconception care strategies to manage preexisting conditions in people with disabilities, interventions to improve their access to outpatient obstetrical and medical care in pregnancy, and resources to prepare them for when ED visits do occur.

## Notes

Supplement 1. **eTable 1. **Description of ICES Datasets Used in the Analyses **eTable 2. **Identification of Recognized Pregnancies **eTable 3. **Identification of Disability **eTable 4. **Risk of Any Emergency Department Visit During Pregnancy, Comparing Individuals With Various Types of Disabilities With Those Without a Disability **eFigure. **Proportion of First Emergency Department Visits by Timing in Pregnancy Comparing Individuals With vs. Without a Disability With a Pregnancy Ending in a Live Birth **eTable 5. **Risk of Any Emergency Department Visit During Pregnancy, Comparing Individuals With Various Types of Disabilities With Those Without a Disability, by Preexisting Comorbidity Status **eTable 6. **Risk of Any Emergency Department Visit During Pregnancy, Comparing Individuals With Various Types of Disabilities With Those Without a Disability, by Pregnancy Outcome **eTable 7. **Risk of Hospital Admission From the Emergency Department Arising During Pregnancy, Comparing Individuals With Various Types of Disabilities With Those Without a Disability **eTable 8. **Outpatient Visit With an Obstetrician or Primary Care Physician Within 7 and 14 Days of an Emergency Department (ED) Visit During Pregnancy, Comparing Individuals With Various Types of Disabilities to Those Without a Disability

Supplement 2.
Data Sharing Statement
