# Supplementary material for: Comparison of Emergency Department Use Between Pregnant People With and Without Disabilities in Ontario, Canada
Source: JAMA Netw Open. 2023 Aug 3;6(8):e2327185. doi: 10.1001/jamanetworkopen.2023.27185 (PMC10401305; doi:10.1001/jamanetworkopen.2023.27185)
Supplement: Supplement 1. — eTable 1. Description of ICES Datasets Used in the Analyses eTable 2. Identification of Recognized Pregnancies eTable 3. Identification of Disability eTable 4. Risk of Any Emergency Department Visit During Pregnancy, Comparing Individuals With Various Types of Disabilities With Those Without a Disability eFigure. Proportion of First Emergency Department Visits by Timing in Pregnancy Comparing Individuals With vs. Without a Disability With a Pregnancy Ending in a Live Birth eTable 5. Risk of Any Emergency Department Visit During Pregnancy, Comparing Individuals With Various Types of Disabilities With Those Without a Disability, by Preexisting Comorbidity Status eTable 6. Risk of Any Emergency Department Visit During Pregnancy, Comparing Individuals With Various Types of Disabilities With Those Without a Disability, by Pregnancy Outcome eTable 7. Risk of Hospital Admission From the Emergency Department Arising During Pregnancy, Comparing Individuals With Various Types of Disabilities With Those Without a Disability eTable 8. Outpatient Visit With an Obstetrician or Primary Care Physician Within 7 and 14 Days of an Emergency Department (ED) Visit During Pregnancy, Comparing Individuals With Various Types of Disabilities to Those Without a Disability [file jamanetwopen-e2327185-s001.pdf]

Supplemental Online Content

Brown HK, Varner C, Ray JG, et al. Comparison of emergency department use between pregnant people with and without disabilities in Ontario, Canada. *JAMA Netw Open*. 2023;6(8):e2327185. doi:10.1001/jamanetworkopen.2023.27185

eTable 1. Description of ICES Datasets Used in the Analyses

eTable 2. Identification of Recognized Pregnancies

eTable 3. Identification of Disability

eTable 4. Risk of Any Emergency Department Visit During Pregnancy, Comparing Individuals With Various Types of Disabilities With Those Without a Disability

eFigure. Proportion of First Emergency Department Visits by Timing in Pregnancy Comparing Individuals With vs. Without a Disability With a Pregnancy Ending in a Live Birth

eTable 5. Risk of Any Emergency Department Visit During Pregnancy, Comparing Individuals With Various Types of Disabilities With Those Without a Disability, by Preexisting Comorbidity Status

eTable 6. Risk of Any Emergency Department Visit During Pregnancy, Comparing Individuals With Various Types of Disabilities With Those Without a Disability, by Pregnancy Outcome

eTable 7. Risk of Hospital Admission From the Emergency Department Arising During Pregnancy, Comparing Individuals With Various Types of Disabilities With Those Without a Disability

eTable 8. Outpatient Visit With an Obstetrician or Primary Care Physician Within 7 and 14 Days of an Emergency Department (ED) Visit During Pregnancy, Comparing Individuals With Various Types of Disabilities With Those Without a Disability

This supplemental material has been provided by the authors to give readers additional information about their work.

© 2023 Brown HK et al. *JAMA Network Open.*

eTable 1. Description of ICES Datasets Used in the Analyses

Data source	Variable	Coding structure	Inception
Canadian Institute for Health Information Discharge Abstract Database	Hospital admissions	Canadian Coding Standards for the International Classification of Diseases and Related Health Problems codes for diagnoses and Canadian Classification of Health Interventions codes for procedures	1988
Census	Sociodemographic data	N/A	2006, 2011, 2016
Immigrants, Refugees, and Citizenship Canada Permanent Residents Database	Immigration status	N/A	1985
National Ambulatory Care Reporting System	Emergency department visits	Canadian Coding Standards for the International Classification of Diseases and Related Health Problems codes for diagnoses and Canadian Classification of Health Interventions codes for procedures	2000
Ontario Health Insurance Database	Outpatient physician visits	Physician billing codes	1991
Ontario Mental Health Reporting System	Psychiatric hospital admissions	Diagnostic and Statistical Manual of Mental Disorders	2005
Registered Persons Database	Sociodemographic data	N/A	1991
Same Day Surgery database	Day surgeries	Canadian Coding Standards for the International Classification of Diseases and Related Health Problems codes for diagnoses and Canadian Classification of Health Interventions codes for procedures	1991

© 2023 Brown HK et al. *JAMA Network Open.*

eTable 2. Identification of Recognized Pregnancies[6]

Pregnancy outcome	Codes	Data sources
Livebirth	m_stillbirth='F'	MOMBABY
Stillbirth	m_stillbirth='T'	MOMBABY
Induced abortion	[ICD-10: O04 or O08] and [CCI: 5CA20FK, 5CA24, 5CA88, or 5CA89, prsuff not in 8, 9]; [OHIP: 635 or 895] and [S785, A920, or P001] and [feesuff = A or B], OR [OHIP: 635 or 895] and [S752] and [feesuff = A or B]	CIHI-DAD, CIHI-SDS, NACRS, OHIP
Miscarriage	[ICD-10: O00, O02.1, or O03]; [OHIP: 632, 633, 634, or 640] and [A920 or P001], OR [A922], OR [OHIP: 632, 633, 634, or 640] and [S752 or S785], OR [S756, S768, S770, or S784]	CIHI-DAD, CIHI-SDS, NACRS OHIP
Threatened abortion	ICD-10: O20; OHIP: 640	CIHI-DAD, CIHI-SDS, NACRS, OHIP

Abbreviations: CCI = Canadian Classification of Health Interventions; CIHI-DAD = Canadian Institute for Health Information Discharge Abstract Database; ICD = International Classification of Diseases and Related Health Problems; OHIP = Ontario Health Insurance Plan; SDS = Same Day Surgery database

© 2023 Brown HK et al. *JAMA Network Open.*

eTable 3. Identification of Disability

Condition name	ICD-10 codes	ICD-9 codes	Other codes
**Physical disability**			
**Congenital anomalies**			
Congenital deformities of the spine	Q67.5	754.2	
Congenital deformities of the feet	Q66	754.5-754.7	OHIP: 754
Congenital musculoskeletal deformities of the chest	Q67.6, Q67.7, Q67.8	754.8	
Dwarfism, not elsewhere classified	E34.3	259.4	
Hypopituitarism	E23.0	253.4	
Other congenital anomalies of the nervous system	Q01.9, Q02-Q04, Q06, Q07.8, Q07.9, G90.1	742	OHIP: 742
Other congenital musculoskeletal deformities	Q75-Q79	756	OHIP: 756
Reduction defects of lower limb	Q72	755.3	
Reduction defects of unspecified limb	Q73, Q74	755.4	
Reduction defects of upper limb	Q71	755.2	
Spina bifida	Q05	741	OHIP: 741
Syndactyly	Q70	755.1	OHIP: 755
**Musculoskeletal disorders**			
Acromegaly and gigantism	E22.0	253.0	
Ankylosing spondylitis	M45, M46	720	OHIP: 720
Chronic osteomyelitis	M86.3-M86.6	730.1	
Disc disorders	M50.0, M50.2-M50.9, M51.0, M51.2-M51.9	722	
Internal derangement of the knee	M22.4, M23.2-M23.5, M23.8, M23.9	717	
Osteoarthritis	M15-M19	715	OHIP: 715
Osteochondropathies	M42, M91, M92, M93	732	OHIP: 732
Osteonecrosis	M87	733.4	
Osteoporosis with history of pathological fracture	M80	733.1	
Polymyalgia rheumatica	M35.3	725	OHIP: 725
Rheumatoid arthritis	M05, M06	714	OHIP: 714
Spondylosis	M47	721	OHIP: 721
**Neurological disorders**			
Cerebral palsy	G80	343	OHIP: 343
Disorders of autonomic nervous system	G90	337	
Epilepsy	G40	345.0-345.1, 345.4-345.9	OHIP: 345
Hemiplegia	G81	342	
Hereditary and idiopathic neuropathy	G60	356	OHIP: 356
Hereditary ataxia and other specified degenerative disorders of the nervous system classified elsewhere	G11, G32.8	334	
Mononeuropathies of the lower limb	G57, G58	355	
Multiple sclerosis	G35	340	OHIP: 340
Muscular dystrophy	G71, G72	359.0	OHIP: 359
Myasthenia gravis	G70	358	OHIP: 358
Nerve root and plexus disorders	G54, G55	353	

© 2023 Brown HK et al. *JAMA Network Open.*

Condition name	ICD-10 codes	ICD-9 codes	Other codes
Other demyelinating diseases of central nervous system	G36, G37	341	
Other disorders of spinal cord	G95	336	
Other extrapyramidal and movement disorders	G10, G23, G24.1-G24.9, G25	333	
Other paralytic syndromes	G82, G83	344	
Other polyneuropathies	G61, G62, G63	357	
Other specified degenerative diseases of the nervous system	G31.8	331.8	
Parkinson's disease	G20, G21	332.0-332.1	OHIP: 332
Sequelae of cardiovascular disease	I69	438	
Sequelae of poliomyelitis	B91	138	
Spinal muscular atrophy and related syndromes	G12	335.1, 335.2, 335.8, 335.9	
**Permanent injuries**			
Brain injury	S02.0, S02.1, S02.3, S02.7-S02.9, S06.1-S06.9, S07, T02.0, T90.5	800.1, 800.3, 801.1, 801.3, 802.6, 802.7, 803.1, 803.3, 804.1, 804.3, 850, 851-854, 907.0, 907.1	
Crushing injury of the lower limb	S77, S87, S97.0, T04.1, T04.3-T04.8	928.0-928.2, 928.8	
Dependence on a wheelchair	Z99.3	V46.3	
Dependence on other enabling machines and devices	Z99.8	V46.8	
Fracture of the lower back or pelvis	S32.4-S32.8, T91.2	808	OHIP: 808
Fracture of the vertebral column with spinal cord injury	S14.0, S14.1, S24.0, S24.1, S34.0, S34.1, S34.3, T06.0, T06.1, T91.3	806, 907.2, 952	OHIP: 806
Other acquired deformities of limbs	M21.8	736.8	
Traumatic amputation of the lower limb	S78, S88, S98.0, S98.3, T05, Z89.4-Z89.8	896, 897, V49.7	
Traumatic amputation of the upper limb	S48, S58, S68.3, S68.4, Z89.1-Z89.3	887, V49.6	
**Sensory disability**			
**Hearing impairments**			
Conductive and sensorineural hearing loss	H90, H91.3, H91.8, H91.9	389	389
Congenital malformations of ear causing impairment of hearing	Q16.0, Q16.1, Q16.3-Q16.9	744.0	
**Vision impairments**			
Blindness and low vision	H54	369	369
Cataracts	H25, H26	366	366
Chorioretinal inflammation	H30, H31	363	
Congenital malformations of the eye	Q11.1, Q11.2, Q13.1, Q13.3, Q13.8, Q15.0	743.0-743.2, 743.4	
Disorders of globe	H44	360	
Disorders of the iris and ciliary body	H20.1	364.1	

© 2023 Brown HK et al. *JAMA Network Open.*

Condition name	ICD-10 codes	ICD-9 codes	Other codes
Disorders of visual cortex	H47.6	377.7	
Glaucoma	H40, H42	365	OHIP: 365
Nystagmus and other irregular eye movements	H55	379.5	
Other retinal disorders	E10.31-E10.35, E11.31-E11.35, H34-H36	362	OHIP: 362
**Intellectual or developmental disabilities**			
Autism and pervasive developmental disabilities	F84.0, F84.1, F84.3-F84.9	299	OHIP: 299; OMHRS: Q2a, Q2b or Q2c (i.e., Axis I) in 299, 299.00, 299.1, 299.10, 299.8, 299.809
Fetal alcohol syndrome	Q86.0		
Intellectual disability	F70-F73, F78, F79	317-319	OHIP: 319; OMHRS: Q2d (i.e., Axis II) in 317, 318, 318.0, 318.1, 318.2, 319 (and retired fields)
Intellectual disability resulting from chromosomal anomalies	Q90, Q91, Q92.0-Q92.5, Q92.7-Q92.9, Q93, Q97.1, Q99.2, Q99.8	758.0-758.3, 758.5, 758.9	OMHRS: I11h–I11m = any diagnosis of Qxxx as listed in ICD-10 column
Other intellectual disabilities (e.g., tuberous sclerosis)	Q85.1, Q86.1, Q87.1, Q87.23, Q87.31, Q87.8	759.5	OMHRS: I11h–I11m = any diagnosis of Qxxx as listed in ICD-10 column; Q3 = 1

© 2023 Brown HK et al. *JAMA Network Open.*

eTable 4. Risk of Any Emergency Department Visit During Pregnancy, Comparing Individuals With Various Types of Disabilities to Those Without a Disability

Disability type	Number (%) with outcome	Unadjusted RR (95% CI)	Adjusted RR (95% CI)[a]	Adjusted RR (95% CI)[b]
No disability (N=2,348,023)	596,771 (25.4)	1.00 (Referent)	1.00 (Referent)	1.00 (Referent)
Physical only (N=221,739)	76,594 (34.5)	1.34 (1.33-1.35)	1.26 (1.25-1.27)	1.24 (1.23-1.24)
Sensory only (N=71,891)	22,421 (31.2)	1.22 (1.20-1.23)	1.15 (1.14-1.17)	1.13 (1.12-1.15)
Intellectual/developmental only (N=3,877)	1,777 (45.8)	1.75 (1.68-1.83)	1.33 (1.28-1.38)	1.28 (1.23-1.33)
Multiple (N=14,359)	6,041 (42.1)	1.62 (1.58-1.65)	1.43 (1.40-1.46)	1.37 (1.34-1.39)

[a] Adjusted for age, parity, neighborhood income quintile, region of residence, immigrant status, stable and unstable chronic conditions, mental illness, and substance use disorders.
[b] Adjusted for age, parity, neighborhood income quintile, region of residence, immigrant status, stable and unstable chronic conditions, mental illness, substance use disorders, continuity of primary care, prenatal care adequacy, and type of prenatal care provider.

© 2023 Brown HK et al. *JAMA Network Open.*

eFigure. Proportion of First Emergency Department Visits by Timing in Pregnancy Comparing Individuals With vs. Without a Disability With a Pregnancy Ending in a Live Birth

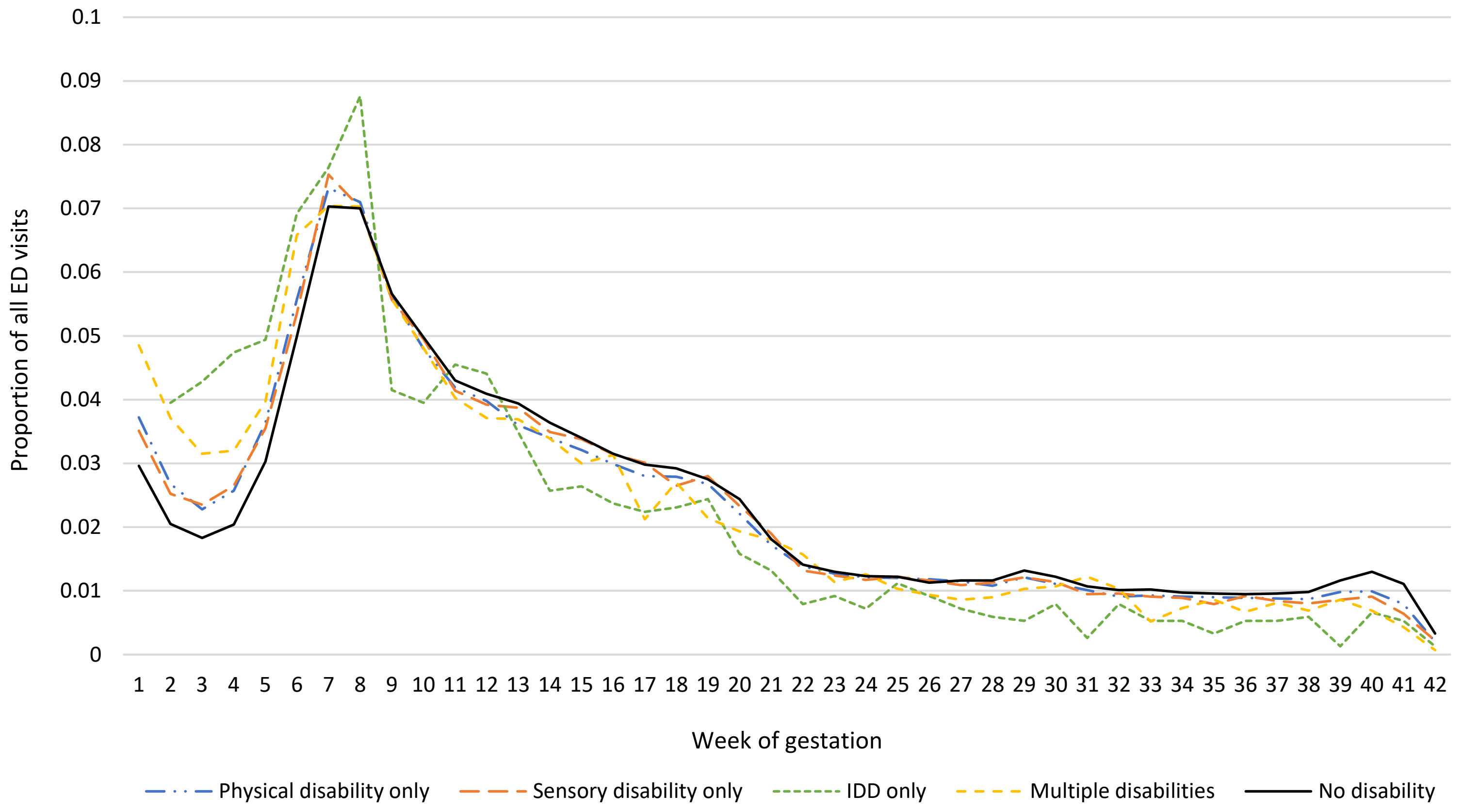

© 2023 Brown HK et al. *JAMA Network Open.*

eTable 5. Risk of Any Emergency Department Visit During Pregnancy, Comparing Individuals With Various Types of Disabilities to Those Without a Disability, by Preexisting Comorbidity Status

Pre-existing comorbidities	Number (%) with outcome	Unadjusted RR (95% CI)	Adjusted RR (95% CI)[b]
**0 pre-existing comorbidities**			
No disability (N=1,455,447)	334,868 (23.0)	1.00 (Referent)	1.00 (Referent)
Physical only (N=113,393)	34,434 (30.4)	1.31 (1.30-1.33)	1.26 (1.25-1.27)
Sensory only (N=39,306)	11,006 (28.0)	1.21 (1.19-1.23)	1.16 (1.14-1.18)
Intellectual/developmental only (N=1,472)	546 (37.1)	1.60 (1.48-1.72)	1.35 (1.26-1.45)
Multiple (N=5,828)	2,088 (35.8)	1.55 (1.49-1.60)	1.44 (1.39-1.49)
**≥ 1 pre-existing comorbidities[a]**			
No disability (N=892,576)	261,903 (29.3)	1.00 (Referent)	1.00 (Referent)
Physical only (N=108,346)	42,160 (38.9)	1.31 (1.30-1.32)	1.25 (1.24-1.27)
Sensory only (N=32,585)	11,415 (35.0)	1.19 (1.17-1.21)	1.14 (1.12-1.16)
Intellectual/developmental only (N=2,405)	1,231 (51.2)	1.70 (1.63-1.78)	1.31 (1.26-1.36)
Multiple (N=8,531)	3,953 (46.3)	1.55 (1.51-1.59)	1.42 (1.39-1.46)

[a] Includes 1 or more stable or unstable chronic condition, mental illness, or substance use disorder.
[b] Adjusted for age, parity, neighborhood income quintile, region of residence, and immigrant status.

© 2023 Brown HK et al. *JAMA Network Open.*

eTable 6. Risk of Any Emergency Department Visit During Pregnancy, Comparing Individuals With Various Types of Disabilities to Those Without a Disability, by Pregnancy Outcome

Pregnancy outcome	Number (%) with outcome	Unadjusted RR (95% CI)	Adjusted RR (95% CI)[a]
**Livebirth**			
No disability (N=1,737,826)	527,191 (30.3)	1.00 (Referent)	1.00 (Referent)
Physical only (N=166,553)	67,335 (40.4)	1.32 (1.31-1.33)	1.25 (1.24-1.25)
Sensory only (N=53,730)	19,839 (36.9)	1.21 (1.19-1.22)	1.14 (1.13-1.16)
Intellectual/developmental only (N=2,909)	1,518 (52.2)	1.67 (1.61-1.74)	1.24 (1.19-1.28)
Multiple (N=10,975)	5,338 (48.6)	1.57 (1.54-1.60)	1.39 (1.36-1.42)
**Stillbirth**			
No disability (N=9,811)	3,916 (39.9)	1.00 (Referent)	1.00 (Referent)
Physical only (N=1,018)	520 (51.1)	1.28 (1.20-1.36)	1.20 (1.12-1.28)
Sensory only (N=333)	150 (45.0)	1.13 (1.00-1.27)	1.08 (0.96-1.22)
Intellectual/developmental only (N=24)	16 (66.7)	1.68 (1.27-2.20)	1.52 (1.17-1.99)
Multiple (N=84)	48 (57.1)	1.43 (1.18-1.72)	1.28 (1.06-1.54)
**Miscarriage**			
No disability (N=98,336)	13,371 (13.6)	1.00 (Referent)	1.00 (Referent)
Physical only (N=10,536)	1,909 (18.1)	1.33 (1.27-1.39)	1.26 (1.21-1.32)
Sensory only (N=3,455)	535 (15.5)	1.14 (1.05-1.23)	1.09 (1.01-1.18)
Intellectual/developmental only (N=167)	49 (29.3)	2.14 (1.68-2.73)	1.59 (1.24-2.03)
Multiple (N=748)	171 (22.9)	1.69 (1.47-1.93)	1.52 (1.34-1.74)
**Induced abortion**			
No disability (N=443,475)	39,028 (8.8)	1.00 (Referent)	1.00 (Referent)
Physical only (N=37,627)	4,942 (13.1)	1.48 (1.44-1.53)	1.36 (1.32-1.39)
Sensory only (N=12,506)	1,391 (11.1)	1.26 (1.20-1.33)	1.14 (1.08-1.20)
Intellectual/developmental only (N=661)	139 (21.0)	2.36 (2.02-2.76)	1.57 (1.35-1.84)
Multiple (N=2,097)	319 (15.2)	1.73 (1.56-1.93)	1.46 (1.32-1.61)
**Threatened abortion**			
No disability (N=58,575)	13,265 (22.6)	1.00 (Referent)	1.00 (Referent)
Physical only (N=6,005)	1,888 (31.4)	1.39 (1.34-1.45)	1.31 (1.26-1.36)
Sensory only (N=1,867)	506 (27.1)	1.20 (1.11-1.29)	1.16 (1.07-1.25)
Intellectual/developmental only (N=116)	55 (47.4)	2.08 (1.71-2.52)	1.45 (1.20-1.75)
Multiple (N=455)	165 (36.3)	1.60 (1.42-1.81)	1.36 (1.20-1.53)

[a] Adjusted for age, parity, neighborhood income quintile, region of residence, immigrant status, stable and unstable chronic conditions, mental illness, and substance use disorders.

© 2023 Brown HK et al. *JAMA Network Open.*

eTable 7. Risk of Hospital Admission From the Emergency Department Arising During Pregnancy, Comparing Individuals With Various Types of Disabilities to Those Without a Disability

Hospital admission from the ED[a]	Number (%) with outcome	Unadjusted RR (95% CI)	Adjusted RR (95% CI)[b]
No disability (N=596,771)	59,796 (10.0)	1.00 (Referent)	1.00 (Referent)
Physical only (N=76,594)	8,579 (11.2)	1.11 (1.08-1.13)	1.08 (1.05-1.10)
Sensory only (N=22,421)	2,009 (9.0)	0.90 (0.86-0.94)	0.89 (0.84-0.93)
Intellectual/developmental only (N=1,777)	241 (13.6)	1.39 (1.22-1.59)	1.23 (1.08-1.40)
Multiple (N=6,041)	750 (12.4)	1.22 (1.13-1.32)	1.16 (1.08-1.25)

[a] Includes only individuals who had an ED visit in pregnancy.
[b] Adjusted for age, parity, neighborhood income quintile, region of residence, immigrant status, stable and unstable chronic conditions, mental illness, and substance use disorders.

eTable 8. Outpatient Visit With an Obstetrician or Primary Care Physician Within 7 and 14 Days of an Emergency Department (ED) Visit During Pregnancy, Comparing Individuals With Various Types of Disabilities to Those Without a Disability

Timing of outpatient visit after initial ED visit	Number (%) with outcome	Unadjusted RR (95% CI)	Adjusted RR (95% CI)[b]
**Within ≤ 7 days[a]**			
No disability (N=573,784)	293,734 (51.2)	1.00 (Referent)	1.00 (Referent)
Physical only (N=74,033)	40,478 (54.7)	1.07 (1.06-1.07)	1.05 (1.05-1.06)
Sensory only (N=21,808)	11,632 (53.3)	1.04 (1.03-1.05)	1.04 (1.03-1.05)
Intellectual/developmental only (N=1,728)	932 (53.9)	1.05 (1.01-1.10)	1.04 (0.99-1.09)
Multiple (N=5,866)	3,446 (58.7)	1.14 (1.12-1.17)	1.11 (1.09-1.14)
**Within ≤ 14 days[a]**			
No disability (N=573,784)	399,543 (69.6)	1.00 (Referent)	1.00 (Referent)
Physical only (N=74,033)	53,880 (72.8)	1.04 (1.04-1.05)	1.04 (1.03-1.04)
Sensory only (N=21,808)	15,621 (71.6)	1.03 (1.02-1.04)	1.03 (1.02-1.04)
Intellectual/developmental only (N=1,728)	1,246 (72.1)	1.04 (1.01-1.07)	1.03 (1.00-1.07)
Multiple (N=6,041)	4,443 (75.7)	1.09 (1.07-1.10)	1.07 (1.05-1.08)

[a] Includes only individuals who had an ED visit during pregnancy.
[b] Adjusted for age, parity, neighborhood income quintile, region of residence, immigrant status, stable and unstable chronic conditions, mental illness, and substance use disorders.

© 2023 Brown HK et al. *JAMA Network Open.*
