# Supplementary material for: Comparison of Emergency Department Use Between Pregnant People With and Without Disabilities in Ontario, Canada
Source: JAMA Netw Open. 2023 Aug 3;6(8):e2327185. doi: 10.1001/jamanetworkopen.2023.27185 (PMC10401305; doi:10.1001/jamanetworkopen.2023.27185)
Supplement: Supplement 2. — Data Sharing Statement [file jamanetwopen-e2327185-s002.pdf]

Data Sharing Statement

Brown. Comparison of Emergency Department Use Between Pregnant People With and Without Disabilities in Ontario, Canada. *JAMA Netw Open*. Published August 03, 2023. doi:10.1001/jamanetworkopen.2023.27185

Data
Data available: No

Additional Information
Explanation for why data not available: Data used for this study were housed at ICES, an independent not-for-profit corporation. While data sharing agreements prohibit ICES from making the data set publicly available, access can be granted to those who meet pre-specified criteria for confidential access, available at www.ices.on.ca/. Requests to access ICES data for research purposes may be submitted to ICES' Data and Analytic Services. Visit http://www.ices.on.ca/DAS for more information, including contact details.
